# Impact of nurse-led anonymous coaching on alcohol craving and motivation to change behaviour among patients with alcohol dependence

**DOI:** 10.6026/973206300212649

**Published:** 2025-08-31

**Authors:** Senthilkumar Azhagirisamy, Venkatesh Mathankumar Vasudevan, Shankar Shanmugam Rajendran, Marudan Anbalagan, Balasubramaniyan Srividhya, Vijayalakshmi Arumugam, Christina Anthonysamy

**Affiliations:** 1 Department of Psychiatric Nursing, College of Nursing, Madras Medical College, The Tamil Nadu Dr. MGR Medical University, Chennai, India; 2 Department of Psychiatry, Institute of Mental Health, Tamil Nadu Dr. MGR Medical University, Chennai, India; 3 Department of Child Health Nursing, College of Nursing, Madras Medical College, Tamil Nadu Dr. MGR Medical University, Chennai, India

**Keywords:** Alcohol dependence, craving, motivation to change, nurse-led coaching, behavioral intervention

## Abstract

Alcohol use disorder (AUD) is a chronic condition marked by compulsive alcohol consumption despite its harmful effects. Harmful alcohol use leads to over 2.6 million deaths annually worldwide. This study assessed the impact of nurse-initiated anonymous coaching sessions on alcohol cravings and motivation to change behavior in alcohol-dependent patients. Over four weeks, 100 participants were divided into experimental and control groups, with the experimental group showing significant reductions in cravings and improvements in motivation. Thus, we show that nurse-led behavioral coaching is an effective, non-pharmacological approach to enhancing AUD recovery.

## Background:

Alcohol dependence, or alcohol use disorder (AUD), is a chronic, recurrent condition marked by an uncontrollable urge to use alcohol despite its adverse impacts on physical health, mental well-being, and social functioning. Globally, detrimental alcohol consumption constitutes a major public health concern [1]. The World Health Organisation indicates that over 2.6 million deaths per year are ascribed to alcohol, or 4.7% of total global fatalities [2]. This challenge highlights the pressing necessity to comprehend and address alcohol abuse proficiently. Alcohol dependence significantly affects physical health. Chronic excessive alcohol consumption harms essential organs, especially the liver, leading to conditions such alcoholic hepatitis and cirrhosis. A systematic analysis indicated that alcohol-related liver illness constitutes approximately 47.9% of all liver-related fatalities [3]. Moreover, prolonged alcohol consumption elevates the risk of cardiovascular diseases, pancreatitis and other malignancies, such as esophageal and liver cancer [4]. The mental health implications are equally alarming; patients with alcohol dependence frequently experience concurrent psychiatric illnesses, including depression, anxiety and cognitive impairments [5]. Studies reveal that 20% to 40% of individuals with Alcohol Use Disorder (AUD) concurrently suffer from at least one comorbid mental health problem, complicating both diagnosis and treatment [6]. The ramifications of alcohol abuse transcend the individual, influencing both familial and societal structures. Families experience emotional distress, financial difficulties and social upheaval as a result of a relative's alcohol abuse. Alcohol is associated with up to 60% of domestic violence incidents, underscoring its societal ramifications [7]. Alcohol misuse incurs an estimated annual economic cost of $249 billion in the United States, primarily attributed to healthcare bills, diminished productivity and criminal justice costs. Young adults and those with a familial history of substance misuse are at increased risk. Adolescents, specifically, experience neurological repercussions, as alcohol consumption can hinder brain growth, leading to subpar academic performance and enduring cognitive difficulties [8]. Approximately 15.1% of young individuals aged 18-24 exhibit binge drinking behaviours, markedly elevating the risk of acquiring dependency [9]. A significant challenge in alcohol rehabilitation is the management of cravings or impulses for alcohol. Intense cravings for alcohol are frequently elicited by environmental stimuli, emotional turmoil, or social contexts. Cravings neurologically stem from modified dopamine transmission and modifications in the brain's reward circuitry, which encourage addictive behaviour [10]. In the recovery process, these desires are particularly acute in the initial phases, elevating the risk of relapse for individuals. Individuals with intense desires are twice as prone to relapse compared to those with minimal desiring intensity [11]. Therefore, it is of interest to assess the impact of nurse-led anonymous coaching on alcohol craving and motivation to change behaviour among patients with alcohol dependence.

## Materials and Methods:

A quasi-experimental, non-randomized control group design was utilised to assess the impact of anonymous nurse-initiated coaching on alcohol cravings and motivation to alter behaviour in individuals with alcohol dependency. The research was carried out over duration of four weeks at the Institute of Mental Health in Chennai, receiving ethical permission from the Institutional Ethics Committee of Madras Medical College, Chennai. A total of 100 patients with alcohol dependence were recruited through a non-probability convenience sampling method. Participants were divided into two groups: experimental (n = 50) and control (n = 50). The inclusion criteria comprised a diagnosis of alcohol dependence (ICD-11), age of 18 years or older, proficiency in Tamil or English, and willingness to provide permission. The exclusion criteria encompassed significant cognitive impairment, concurrent psychiatric disorders, medical instability, or simultaneous involvement in another investigation. After obtaining informed consent, participants completed a pretest consisting of four instruments: (1) Socio-demographic questionnaire, (2) Alcohol Consumption Characteristics Questionnaire, (3) PEN Alcohol Craving Scale (Cronbach's α = 0.89), and (4) Readiness to Change Questionnaire (Cronbach's α = 0.83-0.86). The experimental group underwent organised coaching sessions, whilst the control group received standard care. A post-test with the same instruments was conducted 21 days following the intervention. The experimental group participated in three weekly sessions, each lasting 45 minutes, of anonymous nurse-initiated coaching that included motivational enhancement techniques such as decisional balance, goal setting, and value clarification, as well as distress tolerance exercises like progressive muscle relaxation and paced breathing. These programs were intended to cultivate readiness for change and diminish alcohol cravings. Data were encoded in Microsoft Excel and analysed utilising SPSS version 22. Descriptive statistics were employed for demographic characteristics. Independent t-tests were utilised for between-group comparisons, whereas paired t-tests were employed to analyse within-group differences. Chi-square tests were employed to investigate associations between demographic variables and outcomes. A p-value less than 0.05 were deemed statistically significant.

## Results:

The study enrolled 100 alcohol-dependent patients, evenly divided between experimental and control groups (n = 50 each). The two groups were comparable across all baseline sociodemographic variables, including age, gender, marital status, education, employment, income, and living situation (p > 0.05 for all). Most participants were male (72%), unemployed (40%), and had no formal or only primary education. Daily alcohol consumption was reported by 82% in the experimental group and 70% in the control group. The most commonly consumed beverage was spirits (56% overall). At baseline, there were no statistically significant differences between groups in alcohol urge or eagerness to change behaviour. Clinically significant alcohol cravings were reported by 86% of the experimental group and 88% of the control group (p = 0.77). Regarding motivation to change, 80% in the experimental and 76% in the control group had low eagerness, with no high motivation levels observed (p = 0.62). Following the three-session coaching intervention, the experimental group exhibited marked improvements. In terms of alcohol urge, 24% reported minimal or no craving post-intervention, compared to 0% in the control group. Clinically significant cravings dropped to 32% in the experimental group versus 84% in the control group, a statistically significant difference (χ^2^ = 30.18, p = 0.001). The mean alcohol urge score in the experimental group declined from 23.64 to 17.60, a 20.13% reduction (t = 13.67, p = 0.001), while the control group showed only a 2.4% reduction (from 24.14 to 23.42, p = 0.07). The between-group post-test difference was significant (t = 10.03, p = 0.001) (Table 1 - see PDF). Eagerness to change behavior also improved significantly in the experimental group, with the proportion of highly motivated individuals rising from 0% to 26%. Mean scores increased from 8.28 to 12.90, a 19.25% gain (t = 3.61, p = 0.001). In contrast, the control group showed only a minor improvement (mean score from 8.14 to 8.60; p = 0.19) (Table 2 - see PDF). Significant associations were found between alcohol urge reduction and age (p = 0.01), and living situation (p = 0.05). Participants aged ≥46 and those living alone or with friends had higher residual cravings. Similarly, eagerness to change behavior was significantly influenced by living situation (p = 0.05), duration of alcohol dependence (p = 0.05), and personal recognition of the need to cut down on alcohol (p = 0.01).

## Discussion:

This study assessed the effectiveness of nurse-initiated anonymous coaching sessions in reducing alcohol craving and enhancing motivation to change behavior among alcohol-dependent individuals. The results demonstrated a significant reduction in alcohol urge and a notable improvement in eagerness to change behavior in the experimental group compared to controls, confirming the value of such targeted interventions. Findings align with prior studies suggesting that nurse-initiated anonymous coaching sessions can modulate craving intensity. Weber *et al.* reported that a structured behavioral training program using serious gaming significantly reduced cravings in individuals with alcohol use disorder [12]. Similarly, Korecki *et al.* found mindfulness-based interventions effective in reducing cravings and supporting abstinence through emotional regulation techniques [13]. The intervention also increased motivational readiness. Goldberg *et al.* showed that mindfulness programs not only reduced cravings but significantly improved motivation to sustain recovery efforts [14].

Motivation plays a critical role in behavioral change, and nurse-facilitated coaching in this study incorporated decisional balance and goal-setting tools known to enhance self-efficacy [15]. Comparative analysis of pre- and post-test scores further confirms the program's impact. In the experimental group, motivation scores increased by 19.25%, significantly more than the control group. These results echo findings by Curtiss JE, who showed that combining motivational interviewing with cognitive-behavioral strategies improved drinking behavior outcomes [16]. Moreover, Moreira *et al.* documented that nurse-led brief interventions lowered adolescent alcohol use and increased motivation in a quasi-experimental trial [17]. Participants living with family and those with shorter durations of dependence exhibited better outcomes. Prior research supports this; Moustafa *et al.* demonstrated that intrinsic drinking motives and environmental support strongly influence readiness to change [18]. Similarly, D'Souza and Mathai highlighted demographic and psychosocial predictors of treatment engagement, emphasizing age and insight as key motivators [19]. Willingness to reduce alcohol use was a significant predictor of behavioral change. Zaorska *et al.* showed that distress tolerance is a mediating factor between self-awareness and craving regulation, reinforcing the need for integrated emotional training [20].

## Conclusion:

Nurse-initiated anonymous coaching sessions significantly reduced alcohol cravings and improved motivation for behavioral change in alcohol-dependent patients. Participants in the intervention group demonstrated better outcomes than the control group, with sociodemographic factors influencing treatment effectiveness. Thus, we show integrating nurse-led behavioral coaching into alcohol de-addiction programs for long-term recovery.
